# Surgical Management of Primary and Metastatic Cardiac Malignancies: A 20-Year Single-Center Experience

**DOI:** 10.5761/atcs.oa.26-00025

**Published:** 2026-04-15

**Authors:** Ryo Nakanishi, Hiroto Kawakami, Naoto Tanabe, Koki Tamaoka, Akira Takeuchi, Shoichi Kyo, Tomofumi Taki, Hiroshi Tsuneyoshi

**Affiliations:** Department of Cardiovascular Surgery, Shizuoka General Hospital, Shizuoka, Shizuoka, Japan

**Keywords:** cardiac tumors, cardiac sarcoma, cardiac metastases, surgical oncology, cardiothoracic surgery

## Abstract

**Purpose:**

Malignant cardiac tumors are rare and have a poor prognosis. Surgical resection is performed for symptom relief and survival benefit, but its efficacy is limited. We reviewed our 20-year single-institution experience with primary and metastatic malignant cardiac tumors.

**Methods:**

We retrospectively reviewed 15 patients who underwent resection for histologically confirmed malignant cardiac tumors between 2006 and 2025. Data included tumor characteristics, resection status, and survival outcomes.

**Results:**

Of 15 patients, 7 (46.7%) had primary tumors (most common: angiosarcoma) and 8 (53.3%) had metastatic lesions. The right atrium was the most frequent site (60.0%). Complete (R0) resection was achieved in only 3 cases (20.0%). Median overall survival (OS) for the cohort was 11 months. Patients with angiosarcoma had particularly poor outcomes (median OS: 5 months). One patient with metastatic thymic carcinoma achieved long-term survival (>15 years) following multimodal therapy. Adjuvant therapy was administered in 7 patients (46.7%). No 30-day postoperative mortality occurred.

**Conclusion:**

Surgical resection of malignant cardiac tumors is feasible with acceptable perioperative safety. Although R0 resection is rarely attainable and long-term outcomes remain poor, particularly for high-grade sarcomas, a multimodal approach is essential to optimize outcomes in selected patients.

## Abbreviations

CPB
: cardiopulmonary bypass
RA
: right atrium
UICC
: Union for International Cancer Control

## Introduction

Cardiac tumors can be broadly classified into primary and secondary (metastatic) lesions. Primary tumors originate from the heart itself and are exceedingly rare; autopsy series estimate an incidence of only 0.001%–0.3%, and most (75%–90%) are benign.^1,2)^ Primary malignant tumors, which constitute roughly 10%–25% of primary cardiac lesions, are dominated by sarcomas such as angiosarcoma, primary cardiac lymphomas, and carry a poor prognosis.^1,3)^ Because both primary and metastatic malignancies often present with nonspecific symptoms, diagnosis is frequently delayed, and surgical resection remains the mainstay of therapy and the only potential for cure or meaningful palliation.^4)^ Secondary (metastatic) cardiac tumors arise from lung or breast carcinoma, melanoma, or lymphoma and are far more frequent than primary tumors, forming approximately 95% of all cardiac tumors; these lesions are malignant but are rarely surgically excised.^1)^

Large-scale prospective trials are not feasible for malignant cardiac tumors because of their extreme rarity; therefore, most available evidence is derived from retrospective case series and national database analyses.^5–7)^ A recent multi-institutional analysis of the US National Cancer Database identified more than 100000 cardiac tumors, of which only 826 (0.8%) were primary malignant lesions. Among 747 eligible patients, 59.2% underwent surgical resection. In this cohort, 90-day mortality after surgery alone was 29.4%, while the 30-day, 1-year, and 5-year overall survival (OS) rates were 81.2%, 45.3%, and 11.5%, respectively. Importantly, patients who underwent surgery had significantly better long-term survival compared with those who did not.^5)^

However, several institutional series have reported that complete (R0) resection of primary non-myxomatous cardiac malignancies is rarely achievable, and long-term outcomes remain poor despite aggressive surgical management.^6)^ In particular, studies focusing on primary cardiac sarcomas have shown that angiosarcoma is the most common histologic subtype and that, even with radical resection performed with curative intent (excluding palliative cases) and combined multimodal therapy, median survival is approximately 3 years, with a 5-year survival rate around 30%.^7)^

Given the rarity of such tumors and the limited data on surgical outcomes, we aimed to retrospectively analyze our single-institution experience over the past 20 years with both primary and metastatic malignant cardiac tumors. This study aims to contribute additional evidence to improve surgical management and guide the treatment of these rare and challenging diseases.

## Materials and Methods

### Study design and patient population

This study was a retrospective review of a prospectively maintained surgical database at Shizuoka General Hospital, a tertiary academic medical center. The study was approved by the Institutional Review Board, and the requirement for individual patient consent was waived due to the retrospective nature of the analysis. The study cohort comprised all consecutive patients who underwent surgical intervention for a suspected or pathologically confirmed malignant tumor involving the heart, pericardium, or great vessels between January 1, 2006, and August 31, 2025.

Inclusion criteria were: (1) surgical intervention performed at our institution during the study period, and (2) postoperative pathological confirmation of a primary or metastatic malignant cardiac tumor. Patients with benign cardiac tumors were excluded.

### Data collection and definitions

Data were meticulously extracted from the institutional electronic medical records, surgical operative reports, pathology reports, and outpatient clinic notes. Preoperative diagnosis was established using a combination of imaging modalities, including transthoracic or transesophageal echocardiography, computed tomography (CT), and magnetic resonance imaging (MRI). The collected variables included patient demographics, presenting symptoms, tumor characteristics, surgical approach, operative details, use of cardiopulmonary bypass (CPB), postoperative complications, and adjuvant treatments.

Surgical intent was categorized as either radical or palliative. Resection margins were classified according to the Union for International Cancer Control (UICC) criteria: R0 for no microscopic residual tumor, R1 for microscopic residual tumor, and R2 for macroscopic residual tumor.^8)^

### Endpoints and follow-up

The primary endpoints of the study were 30-day mortality and OS. Thirty-day mortality was defined as death from any cause within 30 days of the index surgical procedure. OS was calculated from the date of surgery to the date of death from any cause or the date of the last known follow-up.

### Statistical analysis

Descriptive statistics were used to summarize patient and treatment characteristics. Continuous variables were presented as median with a range, and categorical variables were reported as counts and percentages. Survival curves were generated using the Kaplan–Meier method. All analyses were performed using EZR (Saitama Medical Center, Jichi Medical University, Saitama, Japan), which is a graphical user interface for R (The R Foundation for Statistical Computing, Vienna, Austria).

## Results

### Patient and tumor characteristics

During the study period, 28 patients were diagnosed with malignant cardiac tumors based on clinical or pathological findings. Of these, 16 underwent surgical intervention. One emergent case was excluded from the analysis due to intraoperative death prior to resection, as the postoperative pathological examination of the pericardial effusion showed only reactive mesothelial cells, failing to confirm malignancy. A final cohort of 15 patients remained. Demographic and tumor characteristics and perioperative findings are summarized in **Table 1**. The median age was 69 years (range, 39–79 years), and 8 patients (53.3%) were male. The most frequent symptoms were dyspnea (n = 5, 33.3%) and chest pain (n = 3, 20.0%). Two patients (13.3%) were asymptomatic at presentation.

**Table 1:** Clinical findings, surgical procedures, and pathology

Pt no.	Age, Sex	Symptoms	Pathology	Size (mm)	Location	Tumor origin	Diagnostic modalities	Approach	Urgency	Surgical intention	Surgery	Concomitant procedure	Postoperative complications	Extent of resection	Immunohistochemical findings
1	72, M	Chest pain	Malignant pericardial mesothelioma	20	Pericardium	Primary	CT: Pericardial nodular structure TTE: Solid echo outside RV free wall	Left thoracotomy	Elective	Drainage of pericardial effusion	Tumor resection and pericardial window	–	–	R0	Positive: Calretinin, D2-40, WT-1, P2 Negative: AE1/AE3, CK5/6, Claudin4
2	77, F	Facial edema and dyspnea on exertion	Invasive thymomic carcinoma	37	SVC-RA	Metastasis	Contrast CT: Suspected SVC tumor or thrombus Transvenous biopsy: Inconclusive (mixed thrombi)	Sternotomy	Elective	Radical resection	Resection and reconstruction of the SVC	Thymoma resection	Right phrenic nerve palsy	R0	–
3	42, M	Abdominal pain	Angiosarcoma	55 × 38 × 20	RA	Primary	Contrast CT: RA tumor TEE was not performed	Sternotomy	Elective	Radical resection	Tumor resection and right atrial reconstruction	–	–	R1	–
4	70, F	Dyspnea	Angiosarcoma	55	RA	Primary	Contrast CT: RA tumor compressing RCA CT-guided biopsy: Suspected leiomyosarcoma TTE: Giant RA tumor	Sternotomy	Elective	Radical resection	Right atrial tumor resection and patch reconstruction, and tricuspid valve repair	–	Dysphagia	R1	Positive: CD31 (diffuse), SMA (focal), c-kit (weak) Negative: desmin, S-100, D2-40, calretinin, CK
5	79, M	Cough	Pulmonary artery sarcoma	41 × 48	PA	Primary	Contrast CT: Tumor within the left pulmonary ligament Bronchoscopy: Negative FDG-PET: Uptake in the left pulmonary artery	Sternotomy	Elective	Radical resection	Left pulmonary artery tumor resection, left pulmonary artery reconstruction	Left pneumonectomy	–	R1	Positive: MDM2, CD34 Negative: desmin, caldesmon, calponin, myogenin
6	63, F	Dyspnea	Metastatic carcinoma (HCC)	60 × 54 × 32	RA	Metastasis	TTE: Giant RA tumor	Sternotomy	Emergent	Prevention of tumor incarceration, pulmonary embolism	Right atrial tumor resection	–	–	R1	–
7	69, F	Dyspnea	Metastasis of high-grade endometrial stromal sarcoma	95 × 80 × 35	RV	Metastasis	Contrast CT and TTE: RA and RV tumor with right ventricular outflow tract (RVOT) obstruction	Sternotomy	Emergent	Relief of heart failure due to incarceration	Right ventricular tumor resection	–	–	R2	Positive: 1A4 (focal), ER, PgR, CD10, c-kit Negative: AE1/AE3+CAM5.2 mix, EMA, desmin, S100, DOG-1
8	39, F	Abdominal pain	Angiosarcoma	70 × 45	RA	Primary	TTE: Sessile tumor on RA free wall. PET: Lymph node and liver metastases VATS biopsy: Angiosarcoma	Sternotomy	Elective	Relief of cardiac tamponade, prevention of sudden death	Right atrial tumor resection, right atrial wall reconstruction, and tricuspid valve repair	–	–	R2	Positive: CD31, CD34
9	52, M	Chest pain	Adenocarcinoma, thymic carcinoma	130 × 115 × 70	Pericardium	Metastasis	CT, MRI, and TTE: Findings of cardiac compression by a pericardial tumor	Sternotomy	Elective	Radical resection	Pericardiectomy	–	–	R2	–
10	69, M	No	Malignant solitary fibrous tumor	51 × 37 × 37	Pericardium	Metastasis	CT (post-mediastinal tumor resection): Findings of cardiac recurrence	Re-Sternotomy	Elective	Radical resection	Pericardiectomy	–	–	R2	–
11	57, F	Dyspnea	Malignant melanoma	80 × 40	RA	Primary	TTE: RA tumor	Sternotomy	Emergent	Prevention of incarceration and heart failure	Right atrial tumor resection	–	–	R2	Positive: S100, HMB45 Negative: CD56, synaptophysin, chromogranin Proliferation index: MIB-1: ca. 60%
12	79, M	Abdominal pain	Intrahepatic cholangiocarcinoma	47	RA, IVC	Metastasis	Contrast CT: Tumor extending from the hepatic vein into the RA	Sternotomy	Emergent	Removal of IVC tumor thrombus	Extended left hepatectomy with resection of tumors involving the inferior vena cava and right atrium	Extended left hepatectomy	Duodenal perforation Acute kidney injury	R1	–
13	74, F	Chest tightness	Angiosarcoma	75 × 45 × 32	RA	Primary	TTE: RA tumor	Sternotomy	Elective	Radical resection	Right atrial tumor resection	–	–	R1	Positive: CD31, CD34, factor VIII
14	74, M	Hoarseness	Thymic carcinoma, squamous cell carcinoma	20 × 10 × 18	Pericardium, mediastinum	Metastasis	CT: Anterior mediastinal tumor noted	Sternotomy	Elective	Radical resection	Anterior mediastinal tumor resection, pericardiectomy	Bentall, CABG (SVG-#2)	Hoarseness and dysphagia	R1	Positive: AE1/AE3, p40, c-kit, PAX-8 Proliferation index: Ki-67: 20%
15	53, M	No	Metastatic adenocarcinoma	55 × 38	RA	Metastasis	Contrast CT: RA tumor noted	Right thoracotomy	Elective	Radical resection	Minimally invasive right atrial tumor resection	–	–	R0	Positive: CK20, CDX2, SATB2 Negative: CK7

Pt, patient; M, male; F, female; HCC, hepatocellular carcinoma; SVC-RA, superior vena cava-right atrium; IVC, inferior venacava; CT, computed tomography; TTE, transthoracic echocardiography; TEE, transesophageal echocardiography; FDG-PET, fluorodeoxyglucose-positron emission tomography; MRI, magnetic resonance imaging; CABG, coronary artery bypass grafting; ER, estrogen receptor, PgR, progesterone receptor

Seven patients (46.7%) were diagnosed with primary cardiac malignancies, and 8 (53.%) had metastatic tumors. Among the primary tumors, angiosarcoma was the most common histology (n = 4), followed by 1 case each of malignant mesothelioma, pulmonary artery sarcoma, and malignant melanoma. The metastatic tumors originated from diverse primary sites, including thymic carcinoma/invasive thymoma (n = 3), adenocarcinoma, hepatocellular carcinoma, endometrial stromal sarcoma, intrahepatic cholangiocarcinoma, and a malignant solitary fibrous tumor. The right atrium (RA) was the most common site of involvement (n = 9, 60.0%).

### Surgical intervention and margin status

**Fig. 1:**
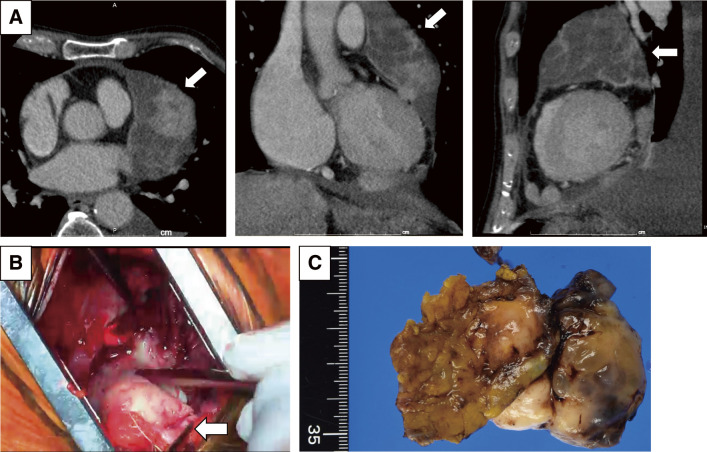
Surgical findings of malignant pericardial mesothelioma. (Patient No. 1) Contrast-enhanced CT demonstrated multiple nodular lesions along the pericardium, suggesting malignant pericardial mesothelioma (panel **A**, arrows). The operation was performed with the patient in the right lateral decubitus position. Through the left fourth intercostal space, the pleura was opened to enter the thoracic cavity. The largest tumor, measuring approximately 5 cm, was excised en bloc with the pericardium (panel **B**). The pericardial defect was enlarged to create a pericardial window. The resected specimen is shown in panel (**C**). CT, computed tomography

**Fig. 2:**
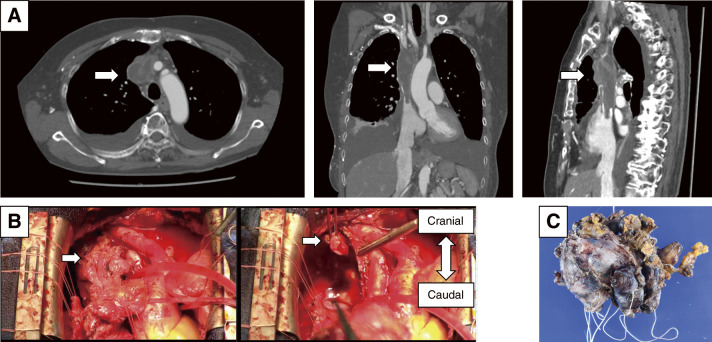
Surgical findings of invasive thymic carcinoma. (Patient No. 2) Contrast-enhanced computed tomography revealed an anterior mediastinal mass and poor opacification of the SVC, which indicated thrombus or bland thrombus (panel **A**, arrows). Intraoperative findings are shown in panel **B**. Median sternotomy was performed, and the anterior mediastinal tumor was carefully dissected. Cardiopulmonary bypass was established via the right femoral artery and right atrium. The tumor within the SVC was completely adherent along its full length (left image; arrow indicates the tumor). The SVC was divided proximally (right image; arrow indicates the SVC). The tumor was resected en bloc (panel **C**). The right phrenic nerve was involved. Reconstruction of the SVC was performed using a PTFE graft. SVC, superior vena cava; PTFE, polytetrafluoroethylene

**Fig. 3:**
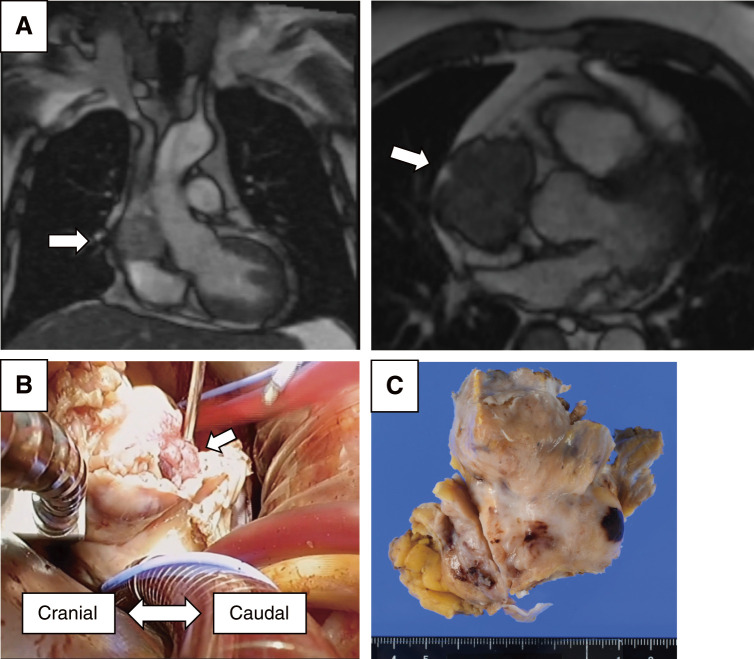
Surgical finding of metastatic adenocarcinoma. (Patient No. 15) Preoperative contrast-enhanced magnetic resonance imaging showed a large mass occupying the right atrium (panel **A**, arrows). Intraoperative findings are shown in panel **B**. Through a right fourth intercostal thoracotomy, the tumor extensively and firmly infiltrated the right atrial wall, extending to the roof of the left atrium. Cardiopulmonary bypass was established via the right femoral artery and vein. The right atrial wall was resected en bloc. A firm, white tumor measuring 55 × 38 mm was completely excised (panel **C**).

The median operative time was 319 min (range, 113–912 min). CPB was utilized in 12 procedures. A median sternotomy was the most frequent approach (n = 13, 86.7%). The surgical intent was radical in 9 cases (60.0%). Despite this, a complete R0 resection was achieved in 3 patients (20.0%). An R1 resection was documented in 7 patients (46.7%), and an R2 resection occurred in 5 patients (33.3%). Reconstruction of the RA or superior/inferior vena cava was performed in 7 cases using a bovine pericardial patch or PTFE graft. The preoperative imaging and intraoperative findings of the 3 patients who achieved R0 resection are presented in **Figs. 1–3** (Patient Nos. 1, 2, and 15).

### Long-term outcomes and survival

**Fig. 4:**
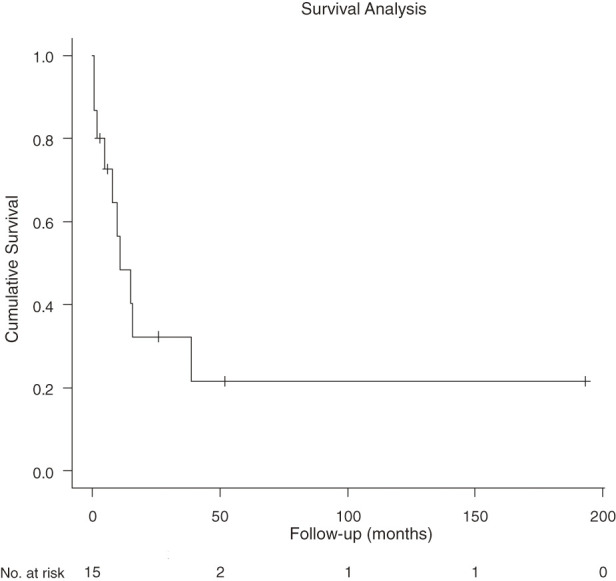
Overall survival for the entire cohort. Kaplan–Meier curve showing overall survival for all 16 patients who underwent a surgical procedure for malignant cardiac tumors. The median overall survival was 11 months (95% CI, 2–39 months). CI, confidence interval

**Fig. 5:**
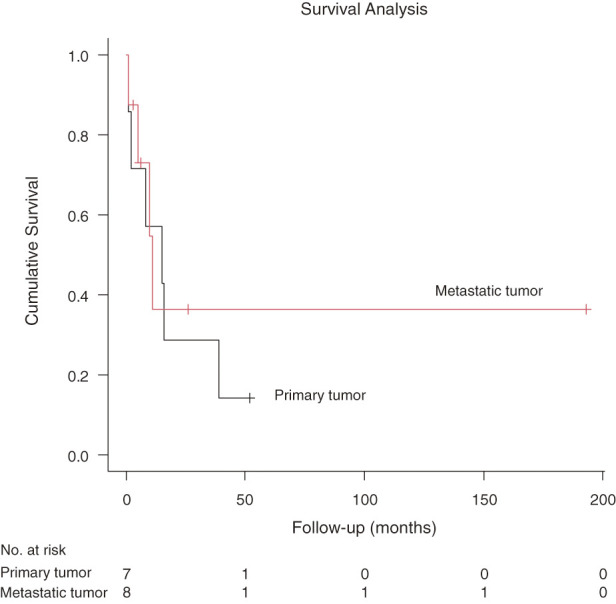
Kaplan–Meier curve comparing overall survival between patients with primary cardiac malignancies (n = 7) and those with metastatic cardiac tumors (n = 8).

**Fig. 6:**
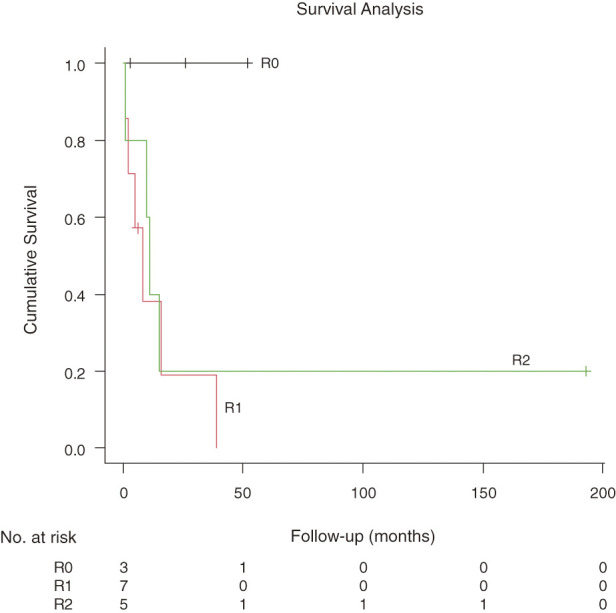
Kaplan–Meier curve comparing overall survival among patients with complete (R0), microscopic residual (R1), and macroscopic residual (R2) resection.

Follow-up was complete for all patients. Perioperative findings, adjuvant therapy, and postoperative follow-up are summarized in **Table 2**. The median length of hospital stay was 15 days (range, 7–151 days). Major postoperative complications occurred in 1 patient (6.7%). All 15 patients who underwent resection survived beyond 30 days postoperatively. The median OS for the entire cohort of 15 patients was 11 months (95% confidence interval, 2–39 months) (**Fig. 4**). When survival was stratified by tumor origin for the 15 resected patients, the median OS was 15 months for primary tumors (n = 7) and 11 months for metastatic tumors (n = 8) (**Fig. 5**). When survival was stratified by resection margin status, the median OS was not reached for the R0 group (n = 3), and the median OS was 8 months for the R1 group (n = 7) and 11 months for the R2 group (n = 5) (**Fig. 6**). When stratified by surgical intent, patients who underwent surgery with radical intent had a median OS of 10 months, whereas those who underwent palliative surgery had a median OS of 5 months.

**Table 2:** Hospital stay and postoperative follow-up

Pt no.	Age, Sex	Pathology	Location	Tumor origin	Length of stay (days)	Discharge status	Status at 30 days	Chemotherapy	Response to chemotherapy	Radiotherapy	Reccurence	Metastasis	Survey	Follow-up (months)
1	72, M	Malignant mesothelioma	Pericardium	Primary	15	Alive	Alive	CBDCA + PEM	Progressive disease	–	Yes	No	Survived	52
2	77, F	Invasive thymoma	SVC-RA	Metastasis	57	Alive	Alive	–	–	–	No	No	Survived	26
3	42, M	Angiosarcoma	RA	Primary	11	Alive	Alive	–	–	–	Yes	Brain	Died	39
4	70, F	Angiosarcoma	RA	Primary	15	Alive	Alive	–	–	50 Gy/25 Fr	No	Multiple bone metastases	Died	8
5	79, M	Pulmonary artery sarcoma	PA	Primary	28	Alive	Alive	–	–	–	Yes	Lung	Died	16
6	63, F	Metastatic carcinoma (HCC)	RA	Metastasis	7	Alive	Alive	–	–	–	Yes	No	Died	1
7	69, F	Metastasis of high-grade endometrial stromal sarcoma	RV	Metastasis	17	Alive	Alive	GEM + DTX	Stable disease	–	No	Brain, lung	Died	11
8	39, F	Angiosarcoma	RA	Primary	8	Alive	Alive	–	–	–	No	Liver, brain	Died	1
9	52, M	Adenocarcinoma, thymic carcinoma	Pericardium	Metastasis	10	Alive	Alive	CBDCA + PTX Pemetrexed	Complete response	Yes (dose was not specified)	No	No	Survived	193
10	69, M	Malignant solitary fibrous tumor	Pericardium	Metastasis	7	Alive	Alive	–	–	48 Gy/16 Fr	Yes	No	Died	10
11	57, F	Malignant melanoma	RA	Primary	15	Alive	Alive	Dacarbazine	Progressive disease	–	Yes	Multiple brain metastases	Died	15
12	79, M	Intrahepatic cholangiocarcinoma	RA, IVC	Metastasis	151	Died	Died	–	–	–	No	No	Died	5
13	74, F	Angiosarcoma	RA	Primary	10	Alive	Alive	–	–	Yes (dose was not specified)	No	No	Died	2
14	74, M	Thymic carcinoma, squamous cell carcinoma	Pericardium, mediastinum	Metastasis	27	Alive	Alive	–	–	–	No	No	Survived	6
15	53, M	Metastatic adenocarcinoma	RA	Metastasis	10	Alive	Alive	–	–	–	No	No	Survived	3

Pt, patient; M, male; F, female; HCC, hepatocellular carcinoma; SVC-RA, superior vena cava-right atrium; IVC, inferior vena cava; CBDCA, carboplatin; PEM, pemetrexed; GEM, gemcitabine; DTX, docetaxel

**Fig. 7:**
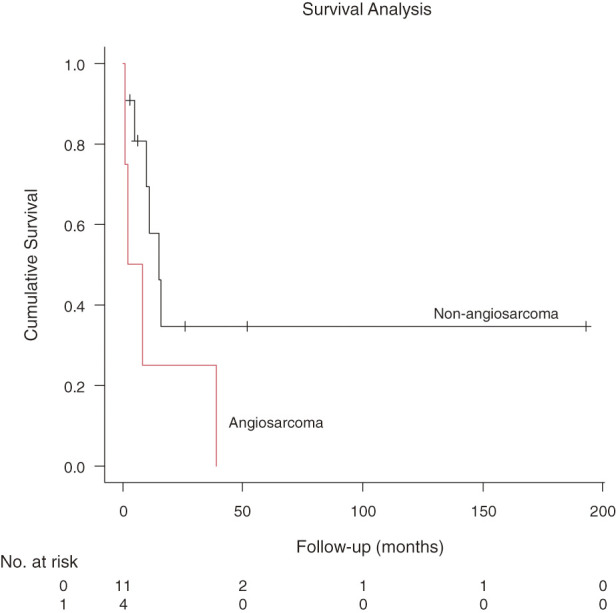
Kaplan–Meier curve comparing overall survival between different histology (angiosarcoma vs. others). The median overall survival of angiosarcoma patients was 5 months (range, 1–39 months).

Survival varied dramatically by histology. The 4 patients with primary angiosarcoma had a median OS of 5 months (**Fig. 7**, range, 1–39 months). In contrast, a patient with metastatic thymic carcinoma achieved long-term survival of 187 months following effective postoperative chemotherapy. Disease recurrence or metastasis was observed in 8 patients (53.3%). Three patients (20.0%) received adjuvant chemotherapy, 3 (20.0%) received radiotherapy, and 1 patient (6.7%) underwent both treatments.

## Discussion

This study highlights the challenges and poor prognosis associated with malignant cardiac tumors, based on a 20-year single-institution surgical experience. Despite aggressive surgical intent, complete (R0) resection was achieved in only 20% of patients, and OS remained poor, with a median of 11 months. Importantly, all resected patients survived beyond 30 days postoperatively, indicating that surgery can be performed safely in selected cases, even in the presence of high surgical complexity.

The low R0 resection rate reflects both the advanced stage at presentation and the anatomic constraints inherent to cardiac tumors. This aligns with previous reports by Boyacıoğlu et al. and Oh et al., where complete excision was also uncommon.^6,8)^ Prior studies consistently demonstrate that R0 resection is a key prognostic factor and should be pursued when feasible, given its association with reduced recurrence and longer survival.^6,9)^ However, due to the limited number of cases in our series, we were unable to demonstrate a statistically significant survival advantage for R0 resection.

In our cohort, all R0 resections were achieved in cases of malignant mesothelioma, invasive thymoma, and colorectal adenocarcinoma. Although primary malignant mesothelioma was categorized as a cardiac primary tumor by our definition, it is more accurately classified as primary malignant pericardial mesothelioma. Therefore, no histologically confirmed primary cardiac malignancy achieved R0 resection in our series. Primary cardiac malignancies often arise within the cardiac chambers and tend to exhibit more diffuse myocardial or endocardial infiltration, making complete resection technically challenging compared with metastatic lesions that involve the pericardial or epicardial surface.^10)^ This observation suggests that tumor origin and pathology influence the feasibility of achieving negative margins. Conversely, the absence of a clear survival difference between primary and metastatic cardiac malignancies in our series may be attributed to the fact that all metastatic cases represented advanced-stage disease at the time of cardiac involvement, thereby offsetting any potential prognostic advantage. Survival varied significantly by histology. Patients with angiosarcoma, the most common primary cardiac sarcoma, experienced particularly poor outcomes, with a median OS of just 5 months—consistent with prior literature describing its aggressive behavior and early metastasis.^7)^

Adjuvant therapy appears to play a crucial role in the treatment strategy. In recent years, neoadjuvant chemotherapy has increasingly been recommended for patients with primary cardiac sarcoma or lymphoma who are hemodynamically stable and have a confirmed tissue diagnosis via biopsy.^11)^ However, in our study, no patient received planned preoperative chemotherapy. Although medical oncologists were consulted for some nonemergency cases, the majority of our patients were symptomatic at the time of presentation; due to the risk of sudden death from tumor embolism, early surgical intervention was prioritized in all cases to achieve immediate symptomatic relief and stabilize the patient’s condition.

In our cohort, about half of the patients received postoperative chemotherapy or radiotherapy. While conclusions are limited by sample size, our findings align with those of Sultan et al. and Hasan et al., who emphasize the survival benefit of multimodal therapy.^5,7)^ These studies support the integration of systemic treatment, even when complete resection is not possible, to improve local control and long-term outcomes. In our study, 1 patient with metastatic thymic carcinoma achieved an exceptional long-term survival of 187 months despite an R2 resection, likely due to a favorable response to postoperative chemoradiotherapy.

Although the distinction between primary and metastatic tumors did not significantly impact survival in our cohort, this may reflect heterogeneity and limited numbers. Histological subtype and responsiveness to adjuvant therapy likely have greater prognostic relevance than tumor origin alone. Our findings suggest that individualized treatment plans, tailored to tumor biology, resectability, and systemic treatment options, are essential.

### Study limitations

This study has several limitations. Its retrospective design is susceptible to selection bias. The small sample size of 15 patients, collected over 2 decades, limits the statistical power of comparative analyses and prevents the development of a reliable multivariable model to identify independent predictors of survival. The long study period introduces heterogeneity in diagnostic imaging, surgical techniques, and the availability and efficacy of adjuvant therapies. Despite these limitations, this study provides a detailed, real-world account of managing these rare diseases from a single center, contributing valuable data to the collective clinical experience.

## Conclusions

Surgical resection of malignant cardiac tumors can be performed with acceptable perioperative outcomes and remains the mainstay of treatment for symptom relief and potential survival benefit. However, complete resection is rarely achievable due to the advanced stage and anatomic limitations of these tumors, and overall prognosis remains poor. Long-term survival may be possible in selected patients—particularly when complete excision is achieved or when effective adjuvant therapy is available. Multimodal management combining surgery, systemic therapy, and radiotherapy should therefore be considered essential. Further multicenter collaboration and prospective data collection are needed to refine surgical strategies and identify patients most likely to benefit from aggressive treatment.

## Declarations

### Data availability

All data generated or analyzed during this study are included in this published article.
